# The Thyroid Na+/I- Symporter: Molecular Characterization and Genomic Regulation

**DOI:** 10.4274/2017.26.suppl.11

**Published:** 2017-01-09

**Authors:** Hani Alotaibi, Merve Tuzlakoğlu-Öztürk, Uygar Halis Tazebay

**Affiliations:** 1 Dokuz Eylül University, Biomedicine and Genome Center (IBG-İzmir), İzmir, Turkey; 2 Gebze Technical University, Department of Molecular Biology and Genetics, Kocaeli, Turkey

**Keywords:** Iodide, Thyroid, Na+/I- symporter, Radiotherapy, thyroid cancer, gene regulation

## Abstract

Iodide (I-) is an essential constituent of the thyroid hormones triiodothyronine (T_3_) and thyroxine (T_4_), and the iodide concentrating mechanism of the thyroid gland is essential for the synthesis of these hormones. In addition, differential uptake of iodine isotopes (radioiodine) is a key modality for the diagnosis and therapy of thyroid cancer. The sodium dependent iodide transport activity of the thyroid gland is mainly attributed to the functional expression of the Na+/I- Symporter (NIS) localized at the basolateral membrane of thyrocytes. In this paper, we review and summarize current data on molecular characterization, on structure and function of NIS protein, as well as on the transcriptional regulation of NIS encoding gene in the thyroid gland. We also propose that a better and more precise understanding of NIS gene regulation at the molecular level in both healthy and malignant thyroid cells may lead to the identification of small molecule candidates. These could then be translated into clinical practice for better induction and more effective modulation of radioiodine uptake in dedifferentiated thyroid cancer cells and in their distant metastatic lesions.

## Abstract (tr)

İyot (I-), tiroid bezi kaynaklı hormonlar olan triiodotironin (T_3_) ve tiroksinin (T_4_) temel bileşenlerindendir, ve tiroid bezinde iyodun derişimini artıran moleküler mekanizmalar aynı zamanda bu hormonların sentezlenmesinde de anahtar rol oynarlar. Bunun yanı sıra, radyoaktif özellikler gösteren iyot izotoplarının normal ve neoplastik dokulara taşınma kinetiklerindeki farklılıklar, bu radyoizotopların tiroid kanserinin teşhis ve tedavisinde etkili olarak kullanılmalarına yol açmıştır. Tiroid bezinde bulunan bu sodium gradyanına eşlenmiş iyot taşıma aktivitesi, temel olarak tiroid folikül hücrelerinin bazolateral hücre zarında bulunan Na+/I- simporter (NIS) proteininin işlevsel ifadesine bağlıdır. Bu derlemede, NIS proteininin yapısı, işlevi ve transkripsiyon ve translasyon sonrası çeşitli moleküler mekanizmalar ile düzenlenmesinin moleküler belirleyicileri özetlenmiştir. Sağlıklı ve kanserleşme özelliği taşıyan tiroid dokularında NIS düzenlenmesinin moleküler detayları daha iyi ve daha detaylı şekilde anlaşıldıkça, hem genetik ve hem de protein düzeyinde NIS’in radyoiyot taşıma etkinliğini düzenleyecek yeni küçük moleküllerin tanımlanabileceğini, bunların klinik uygulamalara aktarılmasının ise özellikle dedifferansiye olmuş tiroid kanseri ve metastazlarının teşhis ve tedavisinde yenilikçi ve etkili uygulamalara yol açacağını öne sürüyoruz.

## INTRODUCTION

## Biological Significance of Iodide Transport

Iodine (^127^I) is the heaviest element metabolized in biological material. It is also a limiting element for the synthesis of covalently bound iodine-containing thyroid hormones, which are essential for proper growth and development of many organs in vertebrates (1,2). Aside from the thyroid gland, which has a remarkable mechanism for collecting and organifying this rare element from nutrients (3), evidence for the presence of iodide (I-) concentrating mechanisms in other vertebrate tissues date back to as early as 1856 when Claude Bernard described the presence of iodine in the salivary gland, in the milk of nursing mothers, as well as in the hair, skin, ovaries, placenta, kidney, stomach and intestines in mammals (4). The availability of radioisotopes by the 1940s improved the techniques in which lower amounts of iodide could be measured, and thus led to precise identification of tissues where iodide transport takes place (4). Also, radioiodide was first used as a therapeutic modality for Graves’ disease, in 1942 at the Massachusetts Institute of Technology (5).

The existence of a mRNA capable of encoding a functional iodide transporter in thyroid cells was illustrated by the expression of a specific mRNA isolated from the thyroid cell line FRTL-5 in the oocytes of X. laevis (6). This study eventually led to cloning of the cDNA of the rat sodium iodide symporter [Na+/I- Symporter, or NIS; (7)]. The cloning of the NIS gene provided an invaluable tool for the analysis of NIS mRNA (and later, protein) expression in different tissues and organs. Since then, while using reverse transcription polymerase chain reaction (RT-PCR) technique, human NIS (hNIS) mRNA was detected in the thyroid, salivary gland, lacrimal ducts, parotid gland, submandibular gland, pituitary gland, pancreas, testis, mammary gland, gastric mucosa, prostate, ovaries, kidney, and placenta (8,9,10,11,12). As expected, the expression of the hNIS mRNA was also clearly observed in both the thyroid and parotid glands, by Northern blot analysis (10).

NIS is the fifth member of the sodium/solute carrier family 5A (SLC5A5; according to the Online Mendelian Inheritance in Man (OMIM) classification). This is a family of proteins that mediate the active transport of negatively-charged solutes (for instance, I-) into the cytoplasm using an electrochemical Na+ gradient (13). The SLC5A5 family of transporters belongs to a solute carrier super-family including 45 different solute carrier families and one organic anion transporter family (14,15). The rat NIS (rNIS) was the first member to be cloned by functional screening of a human cDNA library from FRTL-5, a rat thyroid cell line, in Xenopus laevis oocytes (7). In the same year another report described cloning of the human NIS (hNIS) using cDNA prepared from human papillary carcinoma tissue; they amplified the hNIS cDNA fragment using primers derived from the nucleotide sequence of the rat mRNA of rNIS (16). Subsequently, Tazebay et al. (17) showed that the transport of iodide in lactating mammary glands is also mediated by NIS. Later, the mouse sodium iodide symporter was cloned from the thyroid and lactating mammary gland tissues (18,19).

The gene encoding the human iodide transporter was mapped to chromosomal location 19p13.2-p12 using fluorescence in situ hybridization (8). The coding sequence of the hNIS gene is encoded by 15 exons, and the exon-intron junctions were also clearly described (8).

## Structure of the Na+/I- Symporter

**Figure 1:**
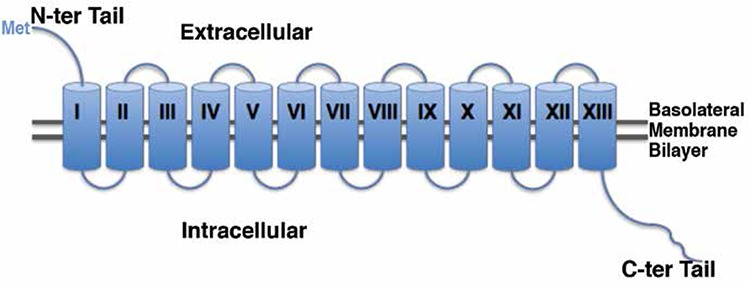
Secondary structure of Na+/I- symporter embedded in basolateral membrane bilayer. Transmembrane domains of Na+/I- symporter crossing the basolateral membrane bilayer are indicated by numbers (I-XIII). The start codon (Met) of Na+/I- symporter is indicated. N-terminal tail of the symporter has an extracellular localization, while the C-terminal part is cytoplasmic. Modified from Levy et al. (22) (1998)

The hNIS cDNA encodes a 643-amino acid protein with 84% homology to both the rat and mouse genes (16). Generation of an anti-NIS antibody allowed researchers to analyze the structure and the post-translational modifications that lead to the mature functional protein. The cytoplasmic location of the carboxy terminus of NIS was confirmed using indirect immunofluorescence in FRTL-5 cells (20). Moreover, by using this antibody, membrane fractions from FRTL-5 cells or COS cells transfected with NIS cDNA revealed a prominent immunoreactive polypeptide with a molecular weight of about 87 kDa, different from the predicted mass of the protein (65 kDa). This difference was attributed to post-translational modifications at three putative Asparagine (Asn) residues at positions 225, 485 and 497 by N-linked glycosylation (20,21), two of which (residues 485 and 497) were located in the predicted sixth extra-cellular loop. Site directed mutagenesis of these putative glycosylation sites demonstrated that NIS is processed at three Asn sites instead of two. This placed the third glycosylation site (previously predicted in the third intracellular loop at position 225) in the cytoplasmic side of the membranesince N-linked glycosylation occurs at exposed extracellular facing sites in the endoplasmic reticulum during protein processing (22). Moreover, by using an amino-terminus FLAG-tagged NIS, engineered by site directed mutagenesis, Levy et al. (22) (1998) showed that non-permeabilized as well as permeabilized cells were stained by anti-FLAG antibodies, an observation suggesting that the amino terminus of NIS is located at the extra-cellular side of the plasma membrane. Based on that, the authors suggested a revised model for the secondary structure of NIS, in which the amino terminus faces extracellularly. According to the current model (Figure 1), NIS is an intrinsic membrane protein, with 13 transmembrane helices (22).

## Function of the Na+/I- Symporter

The sodium/iodide symporter is the transporter responsible for the active transport of iodide from the blood stream into cells. This intracellular iodide is then used in different physiological processes. The uptake process is sodium dependent (23) and NIS co-transports Na+ and I- with a stoichiometry of 2 Na+:1 I- (24). The sodium-driven transport of iodide is maintained by an ouabain sensitive sodium-potassium ATPase which provides the energy required for this process (25,26). NIS is also capable of transporting other ions with less affinity, including ClO_3_-, SCN-, SeCN-, NO_3_-, Br-, BF_4_-, IO_4_-, BrO_3_-, but not perchlorate (ClO_4_-) (24). In fact, ClO_4_- is a well-known competitive inhibitor of iodide transport as well as NO_3_-, BF_4_-, SCN-, 2,4-dinitrophenol, and cardiac glycosides (1,3,24).

## Iodide Requirement in Hormone Biosynthesis and Development

Thyroid hormone biosynthesis requires the presence of inorganic iodide with the presence of an iodide trapping system as the first limiting step in this process (1,3,26). Iodide is an essential and covalently bound constituent of the thyroid hormones triiodothyronine (T_3_) and thyroxin (T_4_). NIS, located at the basolateral membrane of thyrocytes (27), transports iodide into the cells which is then transported across the apical membrane into the follicular lumen (or colloid) by different anion transporters, such as pendrin and apical iodide transporter (28,29). In the colloid, thyroid peroxidase covalently incorporates transported iodide into tyrosine residues of the thyroid hormone precursor, thyroglobulin, in a process known as organification (30,3). Iodinated thyroglobulin is then endocytosed, followed by phagolysosomal hydrolysis of the iodinated thyroglobulin releasing the thyroid hormones, which are then released into the blood stream. This process ismainly controlled by the thyroid stimulating hormone (3). Importantly, thyroid hormones are essential for proper growth and maturation of the skeletal muscles, the nervous system and the lungs of a fetus and a developing newborn (2).

As mentioned before, NIS is also expressed in the lactating mammary glands and functions to secrete iodide into mothers’ milk (17), thus providing the first supply of iodide to the newborn to be used for thyroid hormone biosynthesis. To date, the biological relevance of NIS expression and iodide transport in organs other than the thyroid and the lactating mammary gland is not clear and further research is required to reveal the significance of iodide in the physiological processes in these other organ systems.

## Regulation of Na+/I- Symporter Gene Expression in the Thyroid Gland

In the thyroid gland, thyroid stimulating hormone’s (TSH) binding action elevates the intracellular level of cyclic AMP (cAMP), which, in turn, is an important modulator of gene expression in thyrocytes (31,32). The effect of TSH on iodide uptake in the thyroid was first reported in the 1960s when researchers described increased iodide uptake in the thyroid gland of rats treated with TSH in a cycloheximide dependent manner. This suggested that TSH is actually responsible for the synthesis of an enzyme that mediates iodide uptake (33). Subsequently, by using bovine thyroid cells, Knopp and co-workers confirmed this observation (1970) as well as an inhibitory effect of actinomycin D when added together with TSH. In contrast, cells treated with actinomycin D after two hours of TSH treatment displayed how iodide uptake was stimulated normally, suggesting that TSH treatment resulted in the synthesis of a specific RNA molecule. They also found that cyclohexamide blocked iodide uptake when added with TSH. However, if TSH and cyclohexamide were washed out after two hours, then iodide uptake developed normally (34). In the same study, they also observed a similar effect of TSH when they incubated the cells with cAMP. Cellular levels of cAMP responded to increasing or decreasing concentrations of TSH concluding that TSH in thyroid cells activates adenyl cyclase so that cAMP production is augmented. This increase causes the production of a specific RNA molecule which in turn induces the formation of specific stimulatory protein (34).

These early findings actually suggested a regulatory action of TSH on the expression of NIS in the thyroid gland. Regulation of NIS expression at the transcriptional level was more evident in research studies carried out after the cloning of the NIS gene (7,16,18,19), thus supporting results of earlier reports concerning regulation of NIS expression in thyrocytes. It has been shown that TSH activates the transcription of NIS via cAMP in a cyclohexamide-dependent manner (35). Later on, several reports characterized this TSH stimulated NIS transcription indicating that in the thyroid gland, TSH regulates NIS dependent iodide transport both at post-translational and at transcriptional levels (20,35,36,37,38). Clues for TSH mediated post-translational regulation of NIS came from studies in which membrane vesicles (prepared from FRTL-5 cells that lost iodide uptake as a result of prolonged deprivation of TSH) retained iodide uptake after stimulation by TSH, suggesting that NIS protein is present in the vesicles and a mechanism other than transcription might be required for proper NIS activity (39,37).

The regulatory effect of TSH on NIS protein was illustrated later on; researchers determined that the half-life of NIS protein increases from 3 days to 5 days in the presence of TSH and that NIS is a phosphoprotein, whose phosphorylation is mediated by TSH (37). Moreover, TSH was found to modulate the intra-cellular distribution of NIS; in the presence of TSH, NIS is mainly located at the plasma membrane, whereas in TSH deprived cells, NIS was translocated to intra-cellular compartments (37).

TSH dependent expression of NIS was mediated at the transcriptional levelby the adenylate cyclase-cAMP pathway (3,36). Several groups isolated the 5’ regulatory region of rat and human NIS genes in order to study cis- and trans-acting elements that regulate NIS transcription in thyrocytes (40,41,42,43,36). It was reported that a novel transcription factor, named “NIS TSH-responsive factor-1” or NTF-1, mediates the transcriptional regulatory effect of TSH, itself being mediated by cAMP, on NIS promoter through a TSH responsive element (TRE) located between positions -420 and -385 of the rat NIS promoter in a thyroid specific manner (44).

The thyroid transcription factor-1 [TTF-1; (45)], a member of the Nk2 family of homeobox-containing genes in Drosophila, was implicated in the regulation of several thyroid specific genes such as thyroid peroxidase (TPO), thyroglobulin (Tg) and thyroid stimulating hormone receptor (TSH-R) (46). It was also found to activate the transcription of NIS in thyroid cells, and functional TTF-1 binding sites were found between nucleotide positions -245 to –230 bp of the rNIS promoter (40). Mutations in the NTF-1 binding site (TRE) causing loss of the TSH response also resulted in a decrease in the TTF-1-induced promoter activity, suggesting that TTF-1-mediated thyroid specific expression of NIS is controlled by the TSH/cAMP-pathway (40).

## Sodium-iodide Symporter Upstream Enhancer as a Cis-Regulatory Element

The isolation and cloning of the promoter and upstream regulatory region of the rat NIS gene facilitated the search for cis- and trans-acting genetic elements mediating thyroid-specific and TSH-regulated transcriptional activation (40,41,36). These studies resulted in the identification of a thyroid-specific transcriptional regulatory cis-acting element at the 5’-flanking region of the rat NIS gene (36). This enhancer region (NIS upstream enhancer, or NUE), is located between nucleotides -2495 to -2264 and contains binding sites for Pax8, a paired domain factor that is present both in the thyroid and kidney, and TTF-1, a homeodomain containing protein present in the developing thyroid, lung and diencephalon. In DNase I footprinting studies carried by Ohno et al. (36) (1999), it has been shown that Pax8 actually binds to two sites in this newly identified enhancer. Mutational analysis of these binding sites has shown that Pax8 binding as well as NIS transcription is reduced when these sequences are modified, suggesting a functional role for Pax8 in NUE transcriptional activity. TTF-1 also binds at two different sequences in NUE element, and one of these two binding sites overlaps with Pax8 binding site, while the other is closely located (~20 nucleotides) but distinct from the first site. However, Pax8 (and not TTF-1) is required for the transcriptional activation and cAMP stimulation of the NUE. Further work revealed that a cAMP responsive element (CRE) proximal to NUE can be recognized by various members of the AP-1 and CREB family of transcription factors that modulate the transcriptional activity of NUE. Furthermore, using tethered dimers of b-Zip molecules, it has been shown that specific homo- or hetero-dimers of AP-1 can synergistically stimulate NUE activity in concert with Pax-8 (47). The Human NIS upstream enhancer (hNUE) was also identified; it was found to be localized at –9847 to –8968 bp relative to the hNIS gene start codon. It contains functional Pax8 and TTF-1 binding sites and a CRE-like sequence (48). The enhancer was shown to be cell specific; it activates NIS transcription only in thyroid cell lines, and not in MCF-7 breast cancer or JEG-3 choriocarcinoma cells (48). Recent studies revealed novel transcription factors upregulating NIS transcription in a TSH dependent manner by specific interactions with hNUE; these were β-catenin (49), forkhead transcription factor, FoxE1 (50), and hairy and enhancer of split-1, Hes-1 (51). Also, sterol regulatory element binding proteins (SREBPs) modulate NIS transcription in response to TSH not by interacting with NUE, but with the 5’-UTR of NIS (52).

## Regulation of Na+/I- Symporter by Retinoids in the Thyroid

The ability of thyroid tumors to retain iodide uptake was used for decades for the treatment and diagnosis of thyroid cancer. However, especially in cases with advanced tumorigenesis, thyroid cells suspend this characteristic of radioiodide uptake due to progressive tumor-associated dedifferentiation of thyrocytes, thus leading to a rather ineffective radioiodide therapy [for a review on this, please see (53)]. In earlier reports, it has been shown that in patients with radioiodide resistant tumors, treatment with retinoic acid (RA; a well known agent with differentiation-inducing properties) reactivates the iodide uptake mechanism, and thus restores the possibility of radioiodide-based therapy (54,55). Further characterization of this stimulatory effect of RA in thyroid cell models revealed that RA treatment of normal non-transformed thyrocytes resulted in decreased iodide uptake and reduced NIS expression. On the other hand, both NIS mRNA and iodide uptake were elevated in human follicular thyroid carcinoma cell lines, suggesting that RA treatment could be used to up-regulate NIS expression and thus iodide uptake in tumor cells to be targeted differentially by radioiodide treatment (56).

Retinoic acids are derivatives of vitamin A, which play an important role in several physiological processes during embryonic development and in adult life (57). They are also known for their potent proliferation-inhibiting and differentiation-inducing properties. Retinoic acid signals are mediated by nuclear receptors (Retinoic acid receptors, RAR and Retinoic X receptors, and RXR), action of which can be seen as receptor-receptor interactions, receptor-DNA interactions, andinteractions with other regulatory proteins (57). Transcription activation function of RARs is mediated by binding to DNA sequences called retinoic acid response elements (RARE) in the promoter of target genes (58). The binding site of RARs may vary, depending on the target gene, and the consensus sequence is a hexamer (PuGG/TTCA). The classical RARE is composed of two direct repeats of this core motif, which are usually separated by 5 nucleotides, although functional direct repeats separated by 1, 2 or 10 nucleotides have been also reported (58,59). The molecular determinants controlling RA induced NIS expression in thyroid cells were investigated; RA exerts its up-regulatory effect on hNIS promoter through a RARE located at -1375 relative to the ATG codon (60). It has been shown that RAR binds to this element, specifically DR10 (AGGTCA-n10-GGGTCC), mediating activation of NIS transcription in response to RA stimulation, and that the RA stimulation and RAR binding were abolished due to mutations in either half site of this element (60). This evidence of a direct stimulatory action of RA on NIS expression in thyroid cell lines, as well as the success in RA-redifferentiation prior to radioiodide therapy, encouraged investigators to study also the feasibility of radioiodide therapy after RA treatment in cancer patients with tumors of also other origins (61,62).

## Functional Retinoic Acid Response Elements in Multiple Introns of Na+/I- Symporter Gene

Using the genome Vista software tools (63), the flanking sequences andthe NIS gene were analyzed for conserved regions in the genomes of human, mouse and rat (64). This study was based on the previously shown correlation between conserved gene expression patterns and the conserved regulatory cis-acting elements (65). The results of the analysis revealed several conserved regions which are involved in the regulation of the transcription of this gene. As expected, Alotaibi et al. (64) (2010) first showed that the immediate upstream sequence in front of the NIS transcription start site including the previously described minimal promoter (66) and a RA responsive site (60) responded well to RA in terms of activation of NIS transcription (64). Furthermore, they have demonstrated the significance of intronic sequences in regulating NIS expression: the first intron of NIS contains both DR2-type elements and one overlapping DR10 element, the latter directly interacting with RAR and acting as an enhancer of the gene. Interestingly, multiple introns of NIS gene (7 out-of 14 introns) contain identical functional RARE sequences interacting with the NIS promoter (presumably by formation of a DNA-loop) and regulate the transcriptional activation of the symporter (64). Future molecular studies will determine possible roles of intronic cis-acting nuclear receptor binding sites in organ specific regulation of NIS gene expression.

## Estradiol and ERα are Involved in the Transcription of Na+/I- Symporter

Previous studies have shown that in a rat thyroid cell line model, FRTL-5, activation of ER pathway by E2 down-regulates thyroid NIS gene expression (67,68). Interestingly, in mammary gland cell lines, the RA-dependent expression of NIS was strictly correlated with the presence of ERα, and the analysis of NIS expression in MCF-7 cells where ERα was suppressed by RNA interference revealed the importance of this factor in both basal and tRA induced expression of NIS (69). In the same study, the unliganded ERα (apo-ERα) was shown to play an essential role for holding the NIS gene at a transcriptionally competent state. This would indicate the absence of transcriptionally competent NIS gene loci in ERα- mammary cell lines, and thus explain the lack of tRA-responsive NIS expression in these cells (69). Concerning possibilities of a direct action via ERα, the authors detected a novel ERE sequence conserved in human, rat and mouse genomes in proximity (9 base pairs) of NIS TATA element, with the capacity to activate gene expression in luciferase reporter assays in analyzed ER-positive mammary cell line models (69). In fact, such a close localization of TATA and ERE elements in NIS promoter is very unusual considering that all previously characterized ERE elements were shown to be localized at relatively distant positions to transcription start sites in corresponding genes [although varying remarkably between +23,088 and -2687 (70)].

## Cytokines Modulate Na+/I- Symporter Expression Rather Negatively

Effects of cytokines, such as tumor necrosis factor alpha (TNF-α) and beta (TNF-β), interferon-gamma (IFN-γ), interleukins 1-alpha (IL-1α) and beta (IL-1β), and transforming growth factor-beta (TGF-β) on NIS gene regulation have been assayed by several groups in FRTL-5 cells. All of these factors were found to down-regulate NIS mRNA expression in a time and dose dependent manner (9,71,72,73,74,75,76). Thus it would be correct to state that the effect of cytokines on TSH-induced NIS expression is rather negative. Related with this, there are recent efforts to assess the effectiveness of specific inhibitors of cytokine pathways on an increased NIS expression and radioiodide transport function in advanced thyroid cancers (77).

## Clinical Significance of Iodide Transport in Diagnosis and Therapy of Thyroid Carcinomas

The ability of the thyroid gland to transport iodide is an absolute requirement for the synthesis of T_3_ and T_4_. Significantly, iodide transport mediated by functional NIS expression is also observed in abnormalities of the thyroid such as thyroid nodules and thyroid cancer (78). Thus, the function of NIS -as the key transporter of iodide- has emerged as a valuable tool for the diagnosis and treatment of thyroid cancer, and for decades radioactive iodide played a major therapeutic role in the postoperative management of differentiated thyroid carcinoma (DTC) because of its effectiveness to ablate remnant thyroid tissue and metastases. Moreover, the degree and pattern of iodide accumulation in the thyroid, as revealed by scintigraphic imaging, is used as an aid in the differential diagnosis of thyroid nodules. Thus, radioiodide (^131^I- or ^123^I-) and also pertechnetate (^99m^TcO_4_-) transport activity of NIS have successfully been used in the detection, treatment, and follow-up of thyroid cancers (79).

It has previously been reported in a number of studies that patients with refractory and advanced thyroid cancer may not fully benefit from radioiodide therapy due to insufficient TSH-induced functional NIS expression (16,80,77). By using quantitative RT-PCR techniques, Lazar et al. (81) (1999) have previously shown that NIS mRNA expression was significantly decreased in 40 out of 43 thyroid cancer cases (38 papillary and 5 follicular), and also in about the same percentage of cold adenomas. In the same study, a positive correlation was found between the expression levels of NIS and other thyroid specific proteins such as TPO, Tg, and TSH-R, indicating the link between low expression and dedifferentiation mechanisms. Along the same lines, higher tumor stages were also associated with low NIS expression. Importantly, NIS gene expression levels were also detected to be very low in oncogene-transformed rat thyroid cell models, indicating an inverse correlation between oncogene activation and NIS expression in thyroid cancer models (82). Lower levels of functional NIS expression were also detected in metastatic thyroid tumor tissues. Park et al. (83) (2000) studied correlations in NIS expression between primary thyroid tumors and their metastatic lesions (23 papillary carcinomas, and 7 pairs of primary and lymph node metastatic tissues), indicating variable levels of NIS mRNA expression that were significantly lower than those in healthy thyroid. Furthermore, in a number of cases where NIS expression was detected in the primary tumor site, it was completely absent in their lymph node metastasis, indicating that NIS expression in the primary thyroid tumor could not be used to predict the level of possible radioiodine accumulation in the metastatic lesions.

Retinoic acids (RA) and their derivatives are potent molecules that have been used for redifferentiation therapy of many cancers because of their differentiation-inducing and proliferation-inhibiting abilities (84). They are typically ligands of a class of nuclear receptors called the retinoic acid receptors (RAR and retinoid x receptors; RXR). It has been shown that patients with poorly DTC lacking iodide transport responded to treatments with RA and showed an increased radioiodine transport (54,85,86). It was reasonable to conclude that increased radioiodine transport was a reflection of an increased sodium iodide transporter activity and probably transcription (86). Schmutzler et al. (60) (2002) studied the involvement of RA and RAR in the transcription of NIS using human follicular thyroid carcinoma cell lines and found a dimeric retinoic acid responsive site (DR^10^) at -1375 relative to the ATG start codon of the human NIS gene. Their data showed that this site was responsive to RA stimulation (2.5 fold increase), and that blocking mutations in either half site abolished RAR binding to this element and thus the loss of RA response. Thus, the up-regulatory effect of tRA on thyroid NIS expression was established at the molecular level with several clinical trials successfully demonstrating RA redifferentiation effects in previously dedifferentiated thyroid tumors and their metastases (87,88,89).

## A Few Words on Non-Thyroidal Iodide Transport

Even though the iodide transport function is mostly associated with the thyroid gland, functional expression of NIS has also been reported in healthy mammary gland specifically during late-pregnancy and in lactation (90,17). Interestingly, NIS expression was also observed in a high percentage of human breast cancer specimens (with various pathologies) in contrast to no expression in normal tissues obtained from reductive mammoplasties (17,91). These results suggest that radioiodide administration may be effective as an adjuvant to surgical treatment of primary breast cancer, and/or as a tool in the diagnosis and treatment of metastatic disease (17). A major characteristic of the healthy thyroid gland is that it exhibits NIS activity for life, within boundaries set by thyroid regulatory factors such as thyroid stimulating hormone and iodide itself (74,37). In contrast, the potential effectiveness of radioiodide therapy in breast cancer depends on whether NIS becomes functionally expressed in malignant mammary cells, given that it is not functionally expressed in healthy cells, except during pregnancy and lactation. It is notable that a single transport protein (i.e., NIS) catalyzes the same fundamental process (active Na+-dependent iodide transport) in both tissues, but is regulated differently in each of them. These differences affect not only how NIS functions under normal conditions, but also how it can play a role in cancer management in both tissues.

Unlike the thyroid gland (being the only known organ to incorporate iodide into thyroid proteins), in mammary gland cells iodide is secreted into the milk, and this difference creates a challenge for the application of an effective dose of radioiodide for the treatment of malignant cells of the breast (92). Clearly, alternative strategies for detection of micrometastatic disease and for more effective and targeted systemic therapies are needed to improve survival in breast cancer, which remains the leading cause of cancer deaths in women (ages of 20-59) in developed countries (93).

Several researchers have reported the possible use of radioiodide for the treatment of cancers by forced expression of NIS in tumors of several origins, such as in prostate cancer (94,95), hepatoma (96), glioma (97), neuroendocrine tumor cells (98), head and neck squamous cell carcinoma (99), colon cancer (100,101), pancreatic tumors (102), and in ovarian tumor xenografts (103). The challenge here is the method of delivering NIS expressing DNA selectively to the tumor. So far, studies mentioned above reported the successful use of viral vehicles such as retroviral or adenoviral particles, but more comprehensive studies are certainly required for more effective results, and for a wider range of cancer models to be targeted.

## Conclusion

Thyroid diseases characterized by excess or deficient production of thyroid hormones, enlargement of the gland, presence of aberrant nodules, neoplastic proliferation, and auto-immune syndromes are frequently encountered in endocrinological practice, and thyroid ailments in endocrinology clinics are surpassed in numbers only by diabetes. Since the mid 1940’s, differential expression of thyroid NIS in different pathological conditions leading to a differential biodistribution of iodide isotopes in tissues with different histological and pathological characteristics have made the radioiodide transport system, NIS, a crucial factor for the diagnosis, treatment, or evaluation of pathological thyroid conditions. When NIS is functionally expressed to a sufficient degree in cancerous cells of thyroid origin (and also of mammary glands), use of radioiodide emerges as a powerful potential diagnostic and therapeutic tool. Thus, radioiodide therapy has been used in clinics for decades for the treatment of thyroid cancer, where the goal is to deliver a lethal dose of gamma-radiation to the tumor without affecting the surrounding healthy tissue.

As stated in this paper, a considerable amount of molecular studies has already been carried-out concerning transcriptional regulation of NIS in the thyroid gland, and a substantial amount of studies are being performed to establish novel therapeutic/diagnostic procedures involving small molecules (inhibitors/inducers) and hormonal modalities (TSH, RA, estradiol, etc.) for a refined molecular control of NIS activity in targeted versus untargeted tissues (104), as NIS is expressed not only in thyroid follicular cells but also in lactating breast, gastric mucosa, lacrimal ducts, and salivary ducts. Accordingly, these NIS-expressing tissues are also subject to radioiodine-induced damage during therapeutic procedures. The side effects of radioiodine therapy include temporary or permanent salivary gland disfunction, temporary gastro-intestinal upset, lacrimal duct obstruction, gonadal disfunction, and possible secondary malignancies. Future studies for the molecular protection of non-targeted secondary iodide transporting tissues by, for instance, transiently shutting-down tissue-specific NIS expression will certainly provide an optimal use of radioiodide in the clinical management of thyroid cancer. One of the challenges on this issue is how to have a standard procedure in different cases. This could probably be solved by standardizing the doze of radioiodide, which could be calculated based on the size of the thyroid, the size of the functional part, and with an estimation of metastatic volume. Another challenge is how to protect non-targeted healthy tissue and/or other NIS expressing organs. As mentioned earlier, shutting down the tissue specific expression of NIS from non-targeted organs would be of great benefit, as this also directly affects also the dose received by the specific target tissue (neoplastic target or the metastasis), and thus the effectiveness of radiotherapy.

We also believe that an extensive study of cis- and trans-acting factors regulating the NIS gene in the mammary gland might prove extremely valuable and informative for the efforts of establishing a novel diagnostic and/or therapeutic protocol against lethal breast diseases. In addition, finding candidate small molecules, with molecular effects similar to those of TSH and/or RA in thyroid cells, which could specifically stimulate the functional expression of NIS in other cellular models are also of utmost importance for paving the way for a radioiodide therapy in tissues that do not typically benefit from it.

## Acknowledgements

Research in our laboratories have been generously supported throughout the years by multiple TÜBİTAK grants (101T075; 104T231; 109T049; 109T925; 114Z349 to Uygar Halis Tazebay and 114Z245 to Hani Alotaibi) for which we are grateful.

## References

[1] Wolff J (1964). Transport of iodide and other anions in the thyroid gland. Physiol Rev.

[2] Stubbe P, Schulte FJ, Heidemann P (1986). Iodine deficiency and brain development. Bibl Nutr Dieta.

[3] Carrasco N (1993). Iodide transport in the thyroid gland. Biochim Biophys Acta.

[4] Brown-Grant K (1961). Extrathyroidal iodide concentrating mechanisms. Physiol Rev.

[5] Hertz S, Roberts A, Salter WT (1942). Radioactive iodine as an indicator in thyroid physiology. IV. The metabolism of iodine in Graves’ disease. J Clin Invest.

[6] Vilijn F, Carrasco N (1989). Expression of the thyroid sodium/iodide symporter in Xenopus laevis oocytes. J Biol Chem.

[7] Dai G, Levy O, Carrasco N (1996). Cloning and characterization of the thyroid iodide transporter. Nature.

[8] Smanik PA, Ryu KY, Theil KS, Mazzaferri EL, Jhiang SM (1997). Expression, exon-intron organization, and chromosome mapping of the human sodium iodide symporter. Endocrinology.

[9] Ajjan RA, Kamaruddin NA, Crisp M, Watson PF, Ludgate M, Weetman AP (1998). Regulation and tissue distribution of the human sodium iodide symporter gene. Clin Endocrinol (Oxf).

[10] Spitzweg C, Joba W, Eisenmenger W, Heufelder AE (1998). Analysis of human sodium iodide symporter gene expression in extrathyroidal tissues and cloning of its complementary deoxyribonucleic acids from salivary gland, mammary gland, and gastric mucosa. J Clin Endocrinol Metab.

[11] Mitchell AM, Manley SW, Morris JC, Powell KA, Bergert ER, Mortimer RH (2001). Sodium iodide symporter (NIS) gene expression in human placenta. Placenta.

[12] Spitzweg C, Dutton CM, Castro MR, Bergert ER, Goellner JR, Heufelder AE, Morris JC (2001). Expression of the sodium iodide symporter in human kidney. Kidney Int.

[13] Dohan O, Paroder V, Riedel C, Artani M, Reed M, Ginter CS, Carrasco N (2003). The sodium/iodide symporter (NIS): characterization, regulation, and medical significance. Endocr Rev.

[14] Wain HM, Bruford EA, Lovering RC, Lush MJ, Wright MW, Povey S (2002). Guidelines for human gene nomenclature. Genomics.

[15] Wain HM, Lush MJ, Ducluzeau F, Khodiyar VK, Povey S (2004). Genew: the human gene nomenclature database, 2004 updates. Nucleic Acids Res.

[16] Smanik PA, Liu Q, Furminger TL, Ryu K, Xing S, Mazzaferri EL, Jhiang SM (1996). Cloning of the human sodium lodide symporter. Biochem Biophys Res Commun.

[17] Tazebay UH, Wapnir IL, Levy O, Dohan O, Zuckier LS, Zhao QH, Deng HF, Amenta PS, Fineberg S, Pestell RG, Carrasco N (2000). The mammary gland iodide transporter is expressed during lactation and in breast cancer. Nat Med.

[18] Perron B, Rodriguez AM, Leblanc G, Pourcher T (2001). Cloning of the mouse sodium iodide symporter and its expression in the mammary gland and other tissues. J Endocrinol.

[19] Pinke LA, Dean DS, Bergert ER, Spitzweg C, Dutton CM, Morris JC (2001). Cloning of the mouse sodium iodide symporter. Thyroid.

[20] Levy O, Dai G, Riedel C, Ginter CS, Paul EM, Lebowitz AN, Carrasco N (1997). Characterization of the thyroid Na+/I- symporter with an anti-COOH terminus antibody. Proc Natl Acad Sci USA.

[21] Paire A, Bernier-Valentin F, Selmi-Ruby S, Rousset B (1997). Characterization of the rat thyroid iodide transporter using anti-peptide antibodies. Relationship between its expression and activity. J Biol Chem.

[22] Levy O, Ginter CS, Riedel C, Dai G, Carrasco N (1998). N-linked glycosylation of the thyroid Na+/I- symporter (NIS). Implications for its secondary structure model. J Biol Chem.

[23] Bagchi N, Fawcett DM (1973). Role of sodium ion in active transport of iodide by cultured thyroid cells. Biochim Biophys Acta.

[24] Eskandari S, Loo DD, Dai G, Levy O, Wright EM, Carrasco N (1997). Thyroid Na+/I- symporter. Mechanism, stoichiometry, and specificity. J Biol Chem.

[25] Wolff J, Halmi NS (1963). Thyroidal iodide transport. V. The role of Na-K-activated, ouabain-sensitive adenosinetriphosphatase activity. J Biol Chem.

[26] Baker CH, Morris JC (2004). The sodium-iodide symporter. Curr Drug Targets Immune Endocr Metabol Disord.

[27] Chambard M, Verrier B, Gabrion J, Mauchamp J (1983). Polarization of thyroid cells in culture: evidence for the basolateral localization of the iodide “pump” and of the thyroid-stimulating hormone receptor-adenyl cyclase complex. J Cell Biol.

[28] Bidart JM, Mian C, Lazar V, Russo D, Filetti S, Caillou B, Schlumberger M (2000). Expression of pendrin and the Pendred syndrome (PDS) gene in human thyroid tissues. J Clin Endocrinol Metab.

[29] Rodriguez AM, Perron B, Lacroix L, Caillou B, Leblanc G, Schlumberger M, Bidart JM, Pourcher T (2002). Identification and characterization of a putative human iodide transporter located at the apical membrane of thyrocytes. J Clin Endocrinol Metab.

[30] Igo RP, Mahoney CP, Mackler B (1964). Studies of the biosynthesis of thyroxine. I. Purification and properties of a particulate iodide peroxidase from thyroid tissue. J Biol Chem.

[31] Ikuyama S, Shimura H, Hoeffler JP, Kohn LD (1992). Role of the cyclic adenosine 3’,5’-monophosphate response element in efficient expression of the rat thyrotropin receptor promoter. Mol Endocrinol.

[32] Armstrong R, Wen W, Meinkoth J, Taylor S, Montminy M (1995). A refractory phase in cyclic AMP-responsive transcription requires down regulation of protein kinase A. Mol Cell Biol.

[33] Halmi NS, Granner DK, Doughman DJ, Peters BH, Muller G (1960). Biphasic effect of TSH on thyroidal iodide collection in rats. Endocrinology.

[34] Knopp J, Stolc V, Tong W (1970). Evidence for the induction of iodide transport in bovine thyroid cells treated with thyroid-stimulating hormone or dibutyryl cyclic adenosine 3’,5’-monophosphate. J Biol Chem.

[35] Kogai T, Endo T, Saito T, Miyazaki A, Kawaguchi A, Onaya T (1997). Regulation by thyroid-stimulating hormone of sodium/iodide symporter gene expression and protein levels in FRTL-5 cells. Endocrinology.

[36] Ohno M, Zannini M, Levy O, Carrasco N, di Lauro R (1999). The paired-domain transcription factor Pax8 binds to the upstream enhancer of the rat sodium/iodide symporter gene and participates in both thyroid-specific and cyclic-AMP-dependent transcription. Mol Cell Biol.

[37] Riedel C, Levy O, Carrasco N (2001). Post-transcriptional regulation of the sodium/iodide symporter by thyrotropin. J Biol Chem.

[38] Lauro R, Zannini M (2000). Pax8 has a key role in thyroid cell differentiation. Proc Natl Acad Sci USA.

[39] Kaminsky SM, Levy O, Salvador C, Dai G, Carrasco N (1994). Na(+)-I- symport activity is present in membrane vesicles from thyrotropin-deprived non-I(-)-transporting cultured thyroid cells. Proc Natl Acad Sci USA.

[40] Endo T, Kaneshige M, Nakazato M, Ohmori M, Harii N, Onaya T (1997). Thyroid transcription factor-1 activates the promoter activity of rat thyroid Na+/I- symporter gene. Mol Endocrinol.

[41] Tong Q, Ryu KY, Jhiang SM (1997). Promoter characterization of the rat Na+/I- symporter gene. Biochem Biophys Res Commun.

[42] Behr M, Schmitt TL, Espinoza CR, Loos U (1998). Cloning of a functional promoter of the human sodium/iodide-symporter gene. Biochem J.

[43] Ryu KY, Tong Q, Jhiang SM (1998). Promoter characterization of the human Na+/I- symporter. J Clin Endocrinol Metab.

[44] Ohmori M, Endo T, Harii N, Onaya T (1998). A novel thyroid transcription factor is essential for thyrotropin-induced up-regulation of Na+/I- symporter gene expression. Mol Endocrinol.

[45] Guazzi S, Price M, De Felice M, Damante G, Mattei MG, Lauro R (1990). Thyroid nuclear factor 1 (TTF-1) contains a homeodomain and displays a novel DNA binding specificity. EMBO J.

[46] Damante G, Lauro R (1994). Thyroid-specific gene expression. Biochim Biophys Acta.

[47] Chun JT, Dato V, D’andrea B, Zannini M, Lauro R (2004). The CRE-like element inside the 5’-upstream region of the rat sodium/iodide symporter gene interacts with diverse classes of b-Zip molecules that regulate transcriptional activities through strong synergy with Pax-8. Mol Endocrinol.

[48] Taki K, Kogai T, Kanamoto Y, Hershman JM, Brent GA (2002). A thyroid-specific far-upstream enhancer in the human sodium/iodide symporter gene requires Pax-8 binding and cyclic adenosine 3’,5’-monophosphate response element-like sequence binding proteins for full activity and is differentially regulated in normal and thyroid cancer cells. Mol Endocrinol.

[49] Sastre-Perona A, Santisteban P (2014). Wnt-independent role of β-catenin in thyroid cell proliferation and differentiation. Mol Endocrinol.

[50] Fernandez LP, Lopez-Marquez A, Martinez AM, Gomez-Lopez G, Santisteban P (2013). New insights into FoxE1 functions: identification of direct FoxE1 targets in thyroid cells. PLoS One.

[51] Ferretti E, Tosi E, Po A, Scipioni A, Morisi R, Espinola MS, Russo D, Durante C, Schlumberger M, Screpanti I, Filetti S, Gulino A (2008). Notch signaling is involved in expression of thyrocyte differentiation markers and is down-regulated in thyroid tumors. J Clin Endocrinol Metab.

[52] Ringseis R, Rauer C, Rothe S, Gessner DK, Schütz LM, Luci S, Wen G, Eder K (2013). Sterol regulatory element-binding proteins are regulators of the NIS gene in thyroid cells. Mol Endocrinol.

[53] Brent GA, Kogai T (2013). Cancer: Novel target to enhance radioiodine uptake in thyroid cancer. Nat Rev Endocrinol.

[54] Simon D, Köhrle J, Schmutzler C, Mainz K, Reiners C, Röher HD (1996). Redifferentiation therapy of differentiated thyroid carcinoma with retinoic acid: basics and first clinical results. Exp Clin Endocrinol Diabetes.

[55] Kogai T, Kanamoto Y, Che LH, Taki K, Moatamed F, Schultz JJ, Brent GA (2004). Systemic retinoic acid treatment induces sodium/iodide symporter expression and radioiodide uptake in mouse breast cancer models. Cancer Res.

[56] Schmutzler C, Winzer R, Meissner-Weigl J, Köhrle J (1997). Retinoic acid increases sodium/iodide symporter mRNA levels in human thyroid cancer cell lines and suppresses expression of functional symporter in nontransformed FRTL-5 rat thyroid cells. Biochem Biophys Res Commun.

[57] Pfahl M, Chytil F (1996). Regulation of metabolism by retinoic acid and its nuclear receptors. Annu Rev Nutr.

[58] Giguere V (1994). Retinoic acid receptors and cellular retinoid binding proteins: complex interplay in retinoid signaling. Endocr Rev.

[59] Kato S, Sasaki H, Suzawa M, Masushige S, Tora L, Chambon P, Gronemeyer H (1995). Widely spaced, directly repeated PuGGTCA elements act as promiscuous enhancers for different classes of nuclear receptors. Mol Cell Biol.

[60] Schmutzler C, Schmitt TL, Glaser F, Loos U, Köhrle J (2002). The promoter of the human sodium/iodide-symporter gene responds to retinoic acid. Mol Cell Endocrinol.

[61] Spitzweg C, Scholz IV, Bergert ER, Tindall DJ, Young CY, Göke B, Morris JC (2003). Retinoic acid-induced stimulation of sodium iodide symporter expression and cytotoxicity of radioiodine in prostate cancer cells. Endocrinology.

[62] Abu J, Batuwangala M, Herbert K, Symonds P (2005). Retinoic acid and retinoid receptors: potential chemopreventive and therapeutic role in cervical cancer. Lancet Oncol.

[63] Frazer KA, Pachter L, Poliakov A, Rubin EM, Dubchak I (2004). VISTA: computational tools for comparative genomics. Nucleic Acids Res.

[64] Alotaibi H, Yaman E, Salvatore D, Dato V, Telkoparan P, Lauro R, Tazebay UH (2010). Intronic elements in the Na+/I- symporter gene (NIS) interact with retinoic acid receptors and mediate initiation of transcription. Nucleic Acids Res.

[65] Negre B, Casillas S, Suzanne M, Sanchez-Herrero E, Akam M, Nefedov M, Barbadilla A, Jong P, Ruiz A (2005). Conservation of regulatory sequences and gene expression patterns in the disintegrating Drosophila Hox gene complex. Genome Res.

[66] Venkataraman GM, Yatin M, Ain KB (1998). Cloning of the human sodium-iodide symporter promoter and characterization in a differentiated human thyroid cell line, KAT-50. Thyroid.

[67] Furlanetto TW, Nguyen LQ, Jameson JL (1999). Estradiol increases proliferation and down-regulates the sodium/iodide symporter gene in FTRL-5 cells. Endocrinology.

[68] Kogai T, Schultz JJ, Johnson LS, Huang M, Brent GA (2000). Retinoic acid induces sodium/iodide symporter gene expression and radioiodide uptake in the MCF-7 breast cancer cell line. Proc Natl Acad Sci USA.

[69] Alotaibi H, Yaman EC, Demirpençe E, Tazebay UH (2006). Unliganded estrogen receptor-alpha activates transcription of the mammary gland Na+/I- symporter gene. Biochem Biophys Res Commun.

[70] Bourdeau V, Deschenes J, Metivier R, Nagai Y, Nguyen D, Bretschneider N, Gannon F, White JH, Mader S (2004). Genome-wide identification of high-affinity estrogen response elements in human and mouse. Mol Endocrinol.

[71] Ajjan RA, Watson PF, Findlay C, Metcalfe RA, Crisp M, Ludgate M, Weetman AP (1998). The sodium iodide symporter gene and its regulation by cytokines found in autoimmunity. J Endocrinol.

[72] Pekary AE, Hershman JM (1998). Tumor necrosis factor, ceramide, transforming growth factor-beta1, and aging reduce Na+/I- symporter messenger ribonucleic acid levels in FRTL-5 cells. Endocrinology.

[73] Spitzweg C, Joba W, Morris JC, Heufelder AE (1999). Regulation of sodium iodide symporter gene expression in FRTL-5 rat thyroid cells. Thyroid.

[74] Eng PH, Cardona GR, Fang SL, Previti M, Alex S, Carrasco N, Chin WW, Braverman LE (1999). Escape from the acute Wolff-Chaikoff effect is associated with a decrease in thyroid sodium/iodide symporter messenger ribonucleic acid and protein. Endocrinology.

[75] Eng PH, Cardona GR, Previti MC, Chin WW, Braverman LE (2001). Regulation of the sodium iodide symporter by iodide in FRTL-5 cells. Eur J Endocrinol.

[76] Caturegli P, Hejazi M, Suzuki K, Dohan O, Carrasco N, Kohn LD, Rose NR (2000). Hypothyroidism in transgenic mice expressing IFN-y,gamma in the thyroid. Proc Natl Acad Sci USA.

[77] Lakshmanan A, Scarberry D, Green JA, Zhang X, Selmi-Ruby S, Jhiang SM (2015). Modulation of thyroidal radioiodide uptake by oncological pipeline inhibitors and Apigenin. Oncotarget.

[78] Wollman SH, Reed FE (1960). Radioiodide transport in an iodide-concentrating tumor of thyroid gland. Am J Physiol.

[79] Dadachova E, Carrasco N (2004). The Na/I symporter (NIS): imaging and therapeutic applications. Semin Nucl Med.

[80] Haq M, Harmer C (2004). Thyroid cancer: an overview. Nucl Med Commun.

[81] Lazar V, Bidart JM, Caillou B, Mahe C, Lacroix L, Filetti S, Schlumberger M (1999). Expression of the Na+/I- symporter gene in human thyroid tumors: a comparison study with other thyroid-specific genes. J Clin Endocrinol Metab.

[82] Trapasso F, Iuliano R, Chiefari E, Arturi F, Stella A, Filetti S, Fusco A, Russo D (1999). Iodide symporter gene expression in normal and transformed rat thyroid cells. Eur J Endocrinol.

[83] Park HJ, Kim JY, Park KY, Gong G, Hong SJ, Ahn IM (2000). Expression of human sodium iodide symporter mRNA in primary and metastatic papillary thyroid carcinomas. Thyroid.

[84] Bastien J, Rochette-Egly C (2004). Nuclear retinoid receptors and the transcription of retinoid-target genes. gene.

[85] Simon D, Koehrle J, Reiners C, Boerner AR, Schmutzler C, Mainz K, Goretzki PE, Roeher HD (1998). Redifferentiation therapy with retinoids: therapeutic option for advanced follicular and papillary thyroid carcinoma. World J Surg.

[86] Schmutzler C, Köhrle J (2000). Retinoic acid redifferentiation therapy for thyroid cancer. Thyroid.

[87] Grünwald F, Menzel C, Bender H, Palmedo H, Otte R, Fimmers R, Risse J, Biersack HJ (1998). Redifferentiation therapy-induced radioiodine uptake in thyroid cancer. J Nucl Med.

[88] Koerber C, Schmutzler C, Rendl J, Koehrle J, Griesser H, Simon D, Reiners C (1999). Increased I-131 uptake in local recurrence and distant metastases after second treatment with retinoic acid. Clin Nucl Med.

[89] Simon D, Körber C, Krausch M, Segering J, Groth P, Görges R, Grünwald F, Müller-Gartner HW, Schmutzler C, Köhrle J, Röher HD, Reiners C (2002). Clinical impact of retinoids in redifferentiation therapy of advanced thyroid cancer: final results of a pilot study. Eur J Nucl Med Mol Imaging.

[90] Cho JY, Leveille R, Kao R, Rousset B, Parlow AF, Burak WE, Mazzaferri EL, Jhiang SM (2000). Hormonal regulation of radioiodide uptake activity and Na+/I- symporter expression in mammary glands. J Clin Endocrinol Metab.

[91] Wapnir IL, Nowels K, Amenta PS, Walton K, Montgomery K, Greco RS, Dohan O, Carrasco N (2003). Immunohistochemical profile of the sodium/iodide symporter in thyroid, breast, and other carcinomas using high density tissue microarrays and conventional sections. J Clin Endocrinol Metab.

[92] Zuckier LS, Dadachova E, Dohan O, Carrasco N (2001). The endogenous mammary gland Na(+)/I(-) symporter may mediate effective radioiodide therapy in breast cancer. J Nucl Med.

[93] Greenlee RT, Murray T, Bolden S, Wingo PA (2000). Cancer statistics, 2000. CA Cancer J Clin.

[94] Spitzweg C, O’connor MK, Bergert ER, Tindall DJ, Young CY, Morris JC (2000). Treatment of prostate cancer by radioiodine therapy after tissue-specific expression of the sodium iodide symporter. Cancer Res.

[95] La Perle KM, Shen D, Buckwalter TL, Williams B, Haynam A, Hinkle G, Pozderac R, Capen CC, Jhiang SM (2002). In vivo expression and function of the sodium iodide symporter following gene transfer in the MATLyLu rat model of metastatic prostate cancer. Prostate.

[96] Haberkorn U, Henze M, Altmann A, Jiang S, Morr I, Mahmut M, Peschke P, Kübler W, Debus J, Eisenhut M (2001). Transfer of the human NaI symporter gene enhances iodide uptake in hepatoma cells. J Nucl Med.

[97] Cho JY, Shen DH, Yang W, Williams B, Buckwalter TL, La perle KM, Hinkle G, Pozderac R, Kloos R, Nagaraja HN, Barth RF, Jhiang SM (2002). In vivo imaging and radioiodine therapy following sodium iodide symporter gene transfer in animal model of intracerebral gliomas. Gene Ther.

[98] Schipper ML, Weber A, Behe M, Göke R, Joba W, Schmidt H, Bert T, Simon B, Arnold R, Heufelder AE, Behr TM (2003). Radioiodide treatment after sodium iodide symporter gene transfer is a highly effective therapy in neuroendocrine tumor cells. Cancer Res.

[99] Gaut AW, Niu G, Krager KJ, Graham MM, Trask DK, Domann FE (2004). Genetically targeted radiotherapy of head and neck squamous cell carcinoma using the sodium-iodide symporter (NIS). Head Neck.

[100] Mitrofanova E, Unfer R, Vahanian N, Kane S, Carvour M, Link C (2005). Effective growth arrest of human colon cancer in mice, using rat sodium iodide symporter and radioiodine therapy. Hum Gene Ther.

[101] Scholz IV, Cengic N, Baker CH, Harrington KJ, Maletz K, Bergert ER, Vile R, Göke B, Morris JC, Spitzweg C (2005). Radioiodine therapy of colon cancer following tissue-specific sodium iodide symporter gene transfer. Gene Ther.

[102] Dwyer RM, Bergert ER, O’connor MK, Gendler SJ, Morris JC (2006). Adenovirus-mediated and targeted expression of the sodium-iodide symporter permits in vivo radioiodide imaging and therapy of pancreatic tumors. Hum Gene Ther.

[103] Dwyer RM, Bergert ER, O’connor MK, Gendler SJ, Morris JC (2006). Sodium iodide symporter-mediated radioiodide imaging and therapy of ovarian tumor xenografts in mice. Gene Ther.

[104] Wapnir IL, Goris M, Yudd A, Dohan O, Adelman D, Nowels K, Carrasco N (2004). The Na+/I- symporter mediates iodide uptake in breast cancer metastases and can be selectively down-regulated in the thyroid. Clin Cancer Res.

